# Standard management plan on a mental health ward

**DOI:** 10.1186/2050-2974-3-S1-P22

**Published:** 2015-11-23

**Authors:** Lauren Dasey

**Affiliations:** 1 Gold Coast Hospital and Health Service, Gold Coast, Australia

## 

A consistent and unified approach is paramount to the management of a person being treated for their eating disorder. They require a high level of nursing supervision, support and monitoring, which is often provided by 24 hour one on one nursing care, to assist with containment of compensatory behaviours.

Due to long lengths of stay, and intensive nursing needs, these patients are often looked after by a large number of staff, ranging from ward based staff, to agency staff, experienced and inexperienced staff. Timely access to electronic progress notes and care review summaries can be problematic.

The purpose of this standard management plan is to provide a centralised, paper-based form, which can be individualised to the patient's current level of intervention and monitoring. The form summarises requirements regarding nutrition support, frequency of observations, supervision, mobility and leave. This assists with clinical handover and recognising and responding to clinical deterioration. Both of which are National Safety and Quality Health Service Standards.

The form has also taken the opportunity to include some consumer feedback, as a way of providing information and education to nursing staff who may have limited experience in managing patients with an eating disorder.

